# Level of knowledge and attitude regarding organ donation: a community-based study from Karachi, Pakistan

**DOI:** 10.1186/s13104-019-4345-6

**Published:** 2019-05-30

**Authors:** Farah Khalid, Abdullah Bin Khalid, Danish Muneeb, Asma Shabir, Daniya Fayyaz, Madiha Khan

**Affiliations:** 1 DOW University of Health and Sciences, Karachi, Pakistan; 2 Baqai General Hospital, Karachi, Pakistan; 3 Jinnah Medical University, Karachi, Pakistan

**Keywords:** Organ donation, General population, Knowledge, Awareness, Karachi

## Abstract

**Objective:**

The aim of our study was to assess the knowledge and attitude and to find out the statistics regarding public awareness of organ donation in Karachi. This convenient based, cross-sectional study was conducted from the general population of Karachi, Pakistan from December 2015 to December 2016. The respondents were evaluated through a face to face questionnaire. The questionnaire contained variables regarding knowledge and attitude towards organ donation.

**Result:**

420 people were approached; amongst them 25 refused to participate, so a total number of 395 respondents consented in the research. The mean age of about 77.5% of the population was in the 18–27 range. More than half of the respondents happened to be students and female (51.1%) (55%) respectively. Our results indicate that there was inadequate knowledge among the general population (25.8%). There was a positive attitude regarding organ donation (75.2%). Television was a popular source of information (27%). 29.90% respondents knew that “Kidney” can be donated. 43.80% of the respondents were oblivious to the allowance of organ donation in their religion. More than half (57.2%) were in favor of the promotion of organ donation.

**Electronic supplementary material:**

The online version of this article (10.1186/s13104-019-4345-6) contains supplementary material, which is available to authorized users.

## Introduction

Every year, 6000 patients expire while waiting for an organ donation. Achieving a name on the waiting list means that there is still a 10–30% chance for not getting a transplant. This is due to the scarcity of transplantation organs worldwide [1].

Donation of organs is an imperative component of transplantation [2]. One of the major issues contributing to the scarcity of organs is because of public attributes such as myths, religious misconceptions, and misunderstood decrees [3]. Many previous studies have indicated that knowledge and attitude play a significant role in rates of organ donation [4–6].

The worldwide incidence of knowledge for organ donation varies between 60 and 85%. This change differs from culture and religious beliefs [7]. A study from New York, United States reveled that 88% of people had knowledge about organ donation. Contrastingly, a study conducted in Bursa, Turkey showed that only 60% of Turkish were aware of organ donation [8, 9].

Age, gender, socioeconomic status and education level have been reported to impact attitude towards organ donation [8, 10]; while culture, ethics and religion are also influencing factors [10, 11]. Knowledge about transplants, cadaver and brain death donation has an impact on attitude towards organ donation [12].

Limited research has been carried out in the developing world, where the burden of end-stage organ failure is on the rise and health systems are inadequately equipped [10]. Pakistan is a developing country where 15,000 people are waiting for a kidney transplant while 800 for a liver transplant and 600 for a heart transplant. Two studies were conducted regarding knowledge, attitude, and practice about organ donation. One was conducted in a tertiary care hospital while other targeted general population [10, 12].

## Main text

### Material and methodology

#### Study design and study setting

A cross-sectional study was conducted to evaluate knowledge and attitude of organ donation in general population of Karachi. The study was conducted from December 2015 to December 2016. The target population was the general population of Karachi free of organ failure. The study was conducted in conveniently selected marketplaces from Central, East and South districts of Karachi.

#### Sampling method and sample size

Non-probability, convenience sampling was used in this study. Participants were informed about the questionnaire and were given the proper knowledge about the purpose of the study. A sample size of 385 was used which was calculated by assuming 50% prevalence for attitude and knowledge regarding organ donation, with a confidence level of 95% and 5% sample error [10].

All adults above the age of 18 years belonging to either gender were interviewed after informed consent. Those people who didn’t know the meaning of organ donation were excluded.

#### Method of data collection

Information was collected using a validated questionnaire taken from previous research [10]. Knowledge and attitude of the respondents were evaluated through a face to face questionnaire (Additional file 1).

#### Knowledge and attitude variables

*Knowledge of the respondents* was measured by questions regarding “term organ donation”, “Awareness about Cadaver, Living Donation”, “Allowance of Organ Donation in Religion” and “Risk involved in Organ Donation.”

*The attitude of the respondents* was assessed by questions regarding “Attitude to donate one’s own organ”, “Attitude regarding misuse of organs”, “Promotion of Organ Donation”, “Need of effective laws” and “Attitude regarding donation to a loved one”.

#### Statistical analysis

Data were analyzed by SPSS (Statistical Package Social Sciences). Descriptive statistics, frequency and means were assessed as appropriate. Person Chi-Square test and Fisher Exact test were used to evaluate associations. p-values were studied at < 0.05%.

### Result

420 people were approached, 25 of those refused to participate, so a total number of 395 respondents consented, and filled the questionnaire and thus were included.

#### Demographics

The mean age of about 77.5% of the population fell in 18–27 of range. Most of the respondents were students (51.1%) and female respondents dominated in participation 217 (55%).

#### Knowledge

Following questions were asked regarding knowledge of organ donation.

**Fig. 1:**
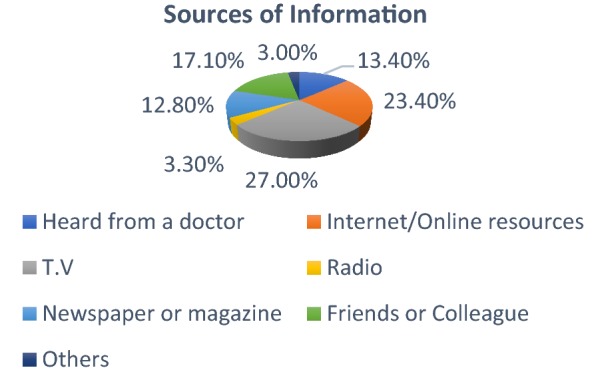
Source of information
- What does “organ donation mean?”
  248 (62.8%) thought that term organ donation meant “Removal of tissue from the human body for transplantation to another person”. 88 (22.3%) responded to the option of “All of the above” which was correct. 21 (5.32%) knew that “Removal of tissue from the human body from a cadaver”.
- What can be the reasons for organ donation?
  81.8% answered that it’s performed “To save someone’s life”. 9.9% population responded, “For money”, 5.6% population selected, “Out of compassion/sympathy”.
- What organs can be donated?
  29.90% knew that “Kidney” can be donated. 21.80% knew about “Eye” donation, however 4.80% knew about “Skin” donation.

- What was your source of Information regarding organ donation?
  27% choose television while 23.40% choose the internet. 3.30% came to know about it from Radio (Fig. 1).

##### Knowledge score

There was low knowledge found among the participants 395 (25.8%). There was no significant association found between level of knowledge and age (p = 0.434), gender (p = 0.450), occupation (p = 0.053), and marital status (p = 0.467). Details are presented in Table 1.

**Table 1:** Knowledge score of organ donation by demographic variables

Demographic variables	Knowledge score of organ donation				
	Inadequate knowledge	%	Adequate knowledge	%	p-value
Age					
18–27	231	75.5	75	24.5	0.412
28–37	56	70.9	23	29.1	
38–45	6	60.0	4	40	
Gender					
Male	131	73.6	47	26.4	0.450
Female	162	74.7	55	25.3	
Occupation					
Student	160	79.2	42	20.8	0.053
Employed	106	67.9	50	32.1	
Unemployed	27	73	10	27	
Marital status					
Single	227	74.4	78	25.6	0.467
Married	66	73.3	24	26.7	
Religion					
Muslim	283	74.3	98	25.7	0.042*
Hindu	9	90.0	1	10.0	
Christian	1	25.0	3	75.0	

* Cell proportion was > 20%

#### Attitude

- Own organs being donated
  106 (26.8%) population selected “Would like to donate nearest/closest one. 87 (22%) chose “Would never consider donating” while 33 (8.4%) stated that they “Would definitely want to donate irrespective of circumstances”.
- Allowance to donate in religion
  173 (43.8%) Majority of the population were unaware of their religion allowing organ donation.
- Donated organs could be misused, abused or misappropriated
  213 (58.5%), stated that donated organs can be misused “Sometimes”. 80 (20.3%) stated organs are misused “Often”, 10 (2.5%) believed that organs are misused “All the time”.
- Whom would you like to donate your organ?”
  227 (57.5%) would donate to a “Family member”, 157 (39.7%) would donate to anyone” while 3 (0.8%) would donate to a “Stranger”.
- An important factor when donating
  175 (44.3%) would donate to a person who is related to them. 114 (28.9%) declared that the health status of the recipient mattered. 73 (18.5%) said that the assurance of respectful treatment of the organs would motivate them, 13 (3.3%) declared “religion of the recipient” will be the most important.
- Consent for living donors
  228 (57.72%) stated “Donor” should give consent himself, while 114 (28.86%) stated family should give consent. 38 (9.62%) believed that “His doctor” should give the consent.
- Consent after death
  “233 (58.9%) believed that family should have the right to give consent, while 93 (23.54%) choose the option of “No one” while 25 (6.33%) opted for the “Doctor”.
- Decisions about organ donation in case of unclaimed dead bodies.
  140 (35.4%) thought that “Medical colleges/doctors” while 114 (28.86%) of the candidates said “no one” should be responsible to make the decision. 86 (21.77%) of the candidates selected “Charitable organizations” while, 41 (10.38%) opted for “A judge”. 14 (3.54%) of the candidates choose “Police”.
- Parents or guardians to make decisions for a mentally disabled person
  The participants gave an equal response to the options “Yes and Don’t know” 137 (34.7%) and 121 (30.6%) stated “No”.
- Promotion of organ donation
  226 (57.2%) people were in “favor” of promotion while 105 (26.6%) selected “don’t know”. 64 (16.2%) were “against” the promotion of organ donation.
- Donated an organ
  379 (95.9%) of a total of 395 participants had never donated any organ while only 16 (4.1%) people had donated an organ.
- Know anyone who donated an organ
  204 (51.6%) didn’t know anyone who has donated an organ. 72 (18.2%) knew someone from the family and 71 (18%) had a friend who has donated an organ.
- Experience of attributes
  226 (57.2%) selected “No”, 136 (34.4%) selected “Don’t know” but only 33 (8.4%) selected option “Yes”.
- Risk of organ donation
  275 (69.6%) selected “Yes”, 17 (4.3%) ticked “No” but only 103 (26.1%) selected “Don’t know”.
- The need for effective laws
  “Yes,” 263 (66.6%) were in favor of the laws. 88 (22.3%) ticked “Don’t know” but only 44 (11.1%) selected “No”.

##### Attitude score

There was a positive attitude regarding organ donation (75.2%). There was no association found between attitude score and age (p = 0.549) and marital status (p = 0.368). But there was an association found between attitude score and gender (p = 0.026), occupation (p = 0.049). Details in Table 2.

**Table 2:** Attitude score of organ donation by demographic variables

Demographic variables	Attitude score of organ donation				
	Inadequate knowledge	%	Adequate knowledge	%	p-value
Age					
18–27	72	23.5	234	76.5	0.549
28–37	23	29.1	56	70.9	
38–45	3	30	7	70	
Gender					
Male	53	29.8	125	70.2	0.026
Female	45	20.7	172	79.3	
Occupation					
Student	41	20.3	161	79.7	0.049
Employed	49	31.4	107	68.6	
Unemployed	8	21.6	29	78.4	
Marital status					
Single	74	24.3	231	75.7	0.368
Married	24	26.7	66	73.3	
Religion					
Muslim	96	25.2	285	74.8	0.478*
Hindu	2	20.0	8	80	
Christian	0	0	4	100	

* Cell proportion was > 20%

### Discussion

This study was conducted to assess the level of knowledge & attitude regarding organ donation among General Population of Karachi, Pakistan. Our findings revealed very interesting points.

Our study shows (25.8%) of the population had adequate knowledge about organ donation while only (22.3%) of them knew the accurate meaning of the process. This can be because we targeted people who were less familiar with the term. Similar was the case with the previous study, but their data showed (60%) prevalence among the public [10].

In a study conducted in Tamil Nadu amongst college students, results showed (28.9%) of the students knew about the meaning [13]. A study conducted in Greece found that health care students were willing to donate and many of them were ready to register, but they weren’t aware of the procedure. In contrary to medical students, British youth was in favor of donation, but few of them were willing to sign up for donation cards whereas they were positive to be the recipient [14].

In a question asked about willingness to donate, our study shows that 8.4% of the population will donate irrespective of circumstances. The result doesn’t match with a previous study that had 32% of the population of undergraduates willing to donate [15].

About 43.8% were unsure regarding acceptance of a donation in their religion which is a shocking dilemma and a study conducted in Chennai highlighted the same problem [3]. In a study conducted in Qatar, no religious disparity was found. But in our study, it was found that about (74.3%) of Muslims think that Islam doesn’t allow organ donation while the Christian community showed positivity for organ donation [16]. Religious, social beliefs, relation to the lifeless body and the sacredness of cadaver prevents from the donation, this was found in research conducted to find the Islamic perspective of organ donation in Pakistan [17].

The kidney was the most popular organ which was known to be donated (29.9%) while eye followed behind (21.8%). This could be because a famous humanitarian of Pakistan, Edhi had donated his cornea after dying. Research conducted in rural areas of Islamabad also showed that the kidney was the most famous organ known to be donated [18]. In another study, cornea donation was found to be a famous donation (96%) [19]. Correspondingly, a study conducted in a tertiary care hospital found corneal donation to be famous [20].

In a study conducted by Brown et al. [21], 57% relatives consented to donate organs of their loved ones. In our study, 57.7% people were allowing the donor to donate his organs if alive, but 59% said that his family should give the consent if dead. Youth in England were also reluctant at donating the organs of their deceased loved ones [14].

Even though Pakistan is a developing country, our study showed that television was an important source of information, the same results were gained from the study conducted in Faisalabad and Islamabad. Similarly, when asked about the promotion of organ donation, 57.2% opted for it same as the previous study [10, 11].

## Strengths

Our study was conducted when philanthropist and humanitarian Abdul Sattar Edhi died. He donated most of his organs plus cornea for transplantation. This event may have played a great role in increasing the knowledge and attitude of the population.

## Limitations

We tried our best to solve issues and present no limitation, but this study only shows attitudes and knowledge of a small population and doesn’t represent masses of Pakistan.

## Additional file

**Additional file 1.** Questionnaire used for collecting data.

## Data Availability

“Not applicable”. All the data is in the result.
