# Supplementary material for: Level of knowledge and attitude regarding organ donation: a community-based study from Karachi, Pakistan
Source: BMC Res Notes. 2019 May 30;12:309. doi: 10.1186/s13104-019-4345-6 (PMC6543638; doi:10.1186/s13104-019-4345-6)
Supplement: Supplementary file 1 — Additional file 1. Questionnaire used for collecting data. [file 13104_2019_4345_MOESM1_ESM.docx]

**APPENDIX**

**Questionnaire**

**Hello, we are the students of Medical Technology and we are conducting the research on the topic AWARENESS OF ORGAN DONATION IN GENERAL POPULATION. This is a questionnaire that will facilitate our research findings by your answers. This surely will keep all the answers confidential. We are grateful for your precious time.**

**Name: __________________________ Age: _______**

**Gender: ________________ Occupation: ________________**

**Marital status: ________________ Residency: _________________**

**Religion: _____________________**

1).Have you ever heard of the term **“Organ Donation”**?

Yes b). No c). Don’t know

2).You heard about **organ donation** through which of the following sources? *(You can choose more than one option)*

a). Heard from a doctor b). Internet /online resources

c). TV d). Radio

e). Newspaper or magazines f). Friend or colleague

g).Other (specify) ____________________________

3).The term **‘Organ Donation’** means?

a).The removal of the [tissues](http://en.wikipedia.org/wiki/Biological_tissue) of the [human body](http://en.wikipedia.org/wiki/Human_body) from a cadaver

b).The removal of the [tissues](http://en.wikipedia.org/wiki/Biological_tissue) of the [human body](http://en.wikipedia.org/wiki/Human_body)from a living donor.

c).The removal of the [tissues](http://en.wikipedia.org/wiki/Biological_tissue) of the [human body](http://en.wikipedia.org/wiki/Human_body)for the purpose of transplantation to another person

d).Can include transfer of cell

e).All of the above

f).Others (specify) _________________________________________________________

4). Why is organ donation done?

a).To save someone’s life

b).Out of compassion/sympathy

c).For money

d).As a ‘responsibility’

e).Others (specify) ________________________________________________________

5).What organs can be donated? (You can choose more than one option)

a). Kidney b). Blood

c). Heart d). Eyes

e). Liver f). Skin

g). Bone marrow h). Lungs

6).On a scale 1-5, your attitude towards the possibility of your own organs being used for **donation?**

**(1=lowest, 5=highest)**

**1**  | **2** | **3** | **4 | 5**

Would never consider donating a donate **(1)**

Will think about it **(2)**

Would like to donate the nearest/closest one **(3)**

Would only like to **donate** under other special circumstances **(4)**

Would definitely want to donate irrespective of circumstances **(5)**

7).Does your religion allow **organ donation?**

a).Yes b). No c).Don‘t know

8).Do you believe that there is a danger that **donated** organs could be misused, abused or misappropriated?

a). Never b). Sometimes c). Often

d). Most of the time e). All the time

9).Who would you like to **donate** your organs to? (*Please pick one option from each set*)

a). Family member b). Stranger c). Friend d). Colleague e). Can be anyone

10).Which of the following factor holds the greatest importance near you when **donating** an organ? (*Choose one option*)

a). Relation to the person b). Age of recipient

c). Religion of recipient d). Health status of recipient

e). Substance abuse of the body f). Assurance of respectful treatment of the organ

11).For **living donation**, who should give consent?

a).Donor b). His family

c).His spouse d). His friends

e).His doctor f). Others (specify) _________________

12).For **donation after death**, who should give consent?

a). No one b). Family

c). Spouse d). Doctor

13).Who should make such decisions about organ donation in case of **unclaimed dead bodies**?

a). Charitable organization b). Medical colleges / doctors c). Police d). A judge e). No one

14).Can parents / guardians make substitute decision making for **mentally disabled** persons in the regard of **organ donation**?

a). Yes b). No c). Don’t know

15).Should **organ donation** be promoted?

a). Yes b). No c). Don’t know

16). Have you ever **donated** an organ?

a). Yes b). No

17). Do you know of anyone who has **donated** an organ?

a). Family member b). Friend c). Colleague d). No one

e).Others (please specify) _______________________________________

18). Have you experienced any effects that you attribute to **organ donation?**

a). Yes b). No c). Don’t know

19). Does **organ donation** involve any risks?

a). Yes b). No c). Don’t know

20). Is there any need for having effective laws to govern the process of **organ donation**?

a). Yes b). No c). Don’t know.

x_____________________x
